# Human Premotor Corticospinal Projections Are Engaged in Motor Preparation at Discrete Time Intervals: A TMS-Induced Virtual Lesion Study

**DOI:** 10.3389/fnrgo.2021.678906

**Published:** 2021-05-19

**Authors:** Robert Fleischmann, Paul Triller, Stephan A. Brandt, Sein H. Schmidt

**Affiliations:** ^1^Vision and Motor System Research Group, Department of Neurology, Charité – Universitätsmedizin Berlin, Berlin, Germany; ^2^Department of Neurology, University Medicine Greifswald, Greifswald, Germany

**Keywords:** transcranial magnetic stimulation, human premotor cortex, navigated brain stimulation, motor preparation, corticospinal tract, virtual lesion

## Abstract

**Objectives:** The significance of pre-motor (PMC) corticospinal projections in a frontoparietal motor network remains elusive. Temporal activation patterns can provide valuable information about a region's engagement in a hierarchical network. Navigated transcranial magnetic stimulation (nTMS)-induced virtual lesions provide an excellent method to study cortical physiology by disrupting ongoing activity at high temporal resolution and anatomical precision. We use nTMS-induced virtual lesions applied during an established behavioral task demanding pre-motor activation to clarify the temporal activation pattern of pre-motor corticospinal projections.

**Materials and Methods:** Ten healthy volunteers participated in the experiment (4 female, mean age 24 ± 2 years, 1 left-handed). NTMS was used to map Brodmann areae 4 and 6 for primary motor (M1) and PMC corticospinal projections. We then determined the stimulator output intensity required to elicit a 1 mV motor evoked potential (1 mV-MT) through M1 nTMS. TMS pulse were randomly delivered at distinct time intervals (40, 60, 80, 100, 120, and 140 ms) at 1 mV-MT intensity to M1, PMC and the DLPFC (dorsolateral pre-frontal cortex; control condition) before participants had to perform major changes of their trajectory of movement during a tracing task. Each participant performed six trials (20 runs per trial). Task performance and contribution of regions under investigation was quantified through calculating the tracing error induced by the stimulation.

**Results:** A pre-motor stimulation hotspot could be identified in all participants (16.3 ± 1.7 mm medial, 18.6 ± 1.4 mm anterior to the M1 hotspot). NTMS over studied regions significantly affected task performance at discrete time intervals (*F*_(10, 80)_ = 3.25, *p* = 0.001). NTMS applied over PMC 120 and 140 ms before changes in movement trajectory impaired task performance significantly more than when applied over M1 (*p* = 0.021 and *p* = 0.003) or DLPFC (*p* = 0.017 and *p* < 0.001). Stimulation intensity did not account for error size (*β* = −0.0074, *p* = 1).

**Conclusions:** We provide novel evidence that the role of pre-motor corticospinal projections extends beyond that of simple corticospinal motor output. Their activation is crucial for task performance early in the stage of motor preparation suggesting a significant role in shaping voluntary movement. Temporal patterns of human pre-motor activation are similar to that observed in intracortical electrophysiological studies in primates.

## Introduction

The human motor system comprises distinct primary (M1) and non-primary motor areas (NPMA) (Rasmussen and Penfield, 1947). While M1 contains prominent pyramidal neurons in layer five and is thus referred to as *area pyramidalis*, more rostral NPMAs undergo a transition to form the so called *areae extrapyramidales* (Foerster, 1936). This has led to the understanding that M1 was the almost exclusive source of corticospinal projections with monosynaptic connections to spinal alpha motoneurons, in particular to hand muscles (Vogt and Vogt, 1926; Schmidlin et al., 2008). Corticofugal projections originating in human NPMAs were considered to consist mainly of brainstem pathways, i.e., the reticulospinal tracts, ultimately projecting onto spinal interneurons and only some corticospinal motoneurons targeting more proximal truncal musculature (Lawrence and Kuypers, 1968; Freund and Hummelsheim, 1985). However, it was shown that NPMAs also contain direct corticospinal motor projections to distal hand muscles (Teitti et al., 2008). We were able to prove that these projections partly originate in the pre-motor cortex (PMC) (Fleischmann et al., 2013). The functional significance of these projections, however, remains to be elucidated.

It is well-established that the PMC is anatomically and functionally embedded in the center of a frontoparietal motor network (Rushworth et al., 2003). It is furthermore understood that it integrates spatial information and tunes the primary motor area for upcoming movements (Thoenissen et al., 2002; Praamstra et al., 2009). This assumption is supported by studies that found PMC lesions to be associated with errors in temporal and spatial coding of movements (Luria, 1966). It could therefore be assumed that pre-motor corticospinal projections would be involved early in human goal directed motor behavior. Yet, alternatively, these projections could be engaged in the same way as M1 corticospinal projections and not pose a distinct entity.

This study aims to clarify the significance of PMC corticospinal projections. Virtual lesions induced by transcranial magnetic stimulation (TMS) can be used to temporally disrupt ongoing activity in a region of interest and thus help understand whether and, if applied at different time intervals, when it is involved in a task (Pascual-Leone et al., 1999). We combined the virtual lesion capability of TMS with navigation technology to differentiate the involvement of M1 and PMC corticospinal projections in a motor task with high temporal and spatial resolution. To exclude an unspecific disruption of task execution through frontal lobe stimulation, we also performed dorsolateral pre-frontal cortical stimulation as control condition.

## Materials and Methods

All procedures involving human participants were conducted in accordance with the ethical standards of the institutional research committee and with the 1964 Helsinki declaration and its later amendments. Formal consent from the institutional review board was obtained.

### Participants and Design

Ten healthy subjects volunteered to participate in this study (4 female, mean age 24 ± 2 years, 1 left-handed). All subjects gave written informed consent before any data was obtained and participants were free to withdraw without reason at any time. Handedness was confirmed by the Edinburgh handedness inventory. A detailed medical history was taken to exclude neurological or psychiatric illness and the presence of implanted electronic devices or ferromagnetic metals.

The experimental procedure was separated in two parts to avoid fatigue effects. During the first session, M1 and PMC stimulation hotspots were mapped (see section Identification of M1 and PMC Stimulation Hotspots) and stored to be used as targets for nTMS virtual lesion induction in the subsequent experimental session (see section Motor Task and Virtual Lesion Induction). Both sessions were performed in the morning to avoid interference from circadian fluctuations in corticospinal excitability.

### Navigated Transcranial Magnetic Stimulation

Individual structural MRI (3D-MPRAGE, matrix 256 × 256, 180 sagittal slices, voxel size 1 mm^3^, on a GE 3 Tesla scanner) were acquired. The subject's head was tracked by an infrared-based stereotactical system and brought into co-registration with the MRI using a triangular system of anatomical landmarks (bilateral tragus and nasion) as well as a subsequent 9-point surface registration. The eXimia system calculates the strength, location and direction of the stimulating electric field in the cortical tissue based on a dynamic, real-time adjusted spherical model, that takes individual head size and shape as well as the physical parameters of stimulation into account (Ilmoniemi et al., 1999). TMS pulses were delivered through an eXimia TMS stimulator connected to a focal monophasic figure-of-eight coil (70 mm outer diameter) (Nexstim, Helsinki, Finland). The precision of nTMS is considered to be comparable to that of intraoperative direct cortical stimulation (Picht et al., 2009). Surface electrodes were attached over the first dorsal interosseous (FDI) of the subject's dominant hand, using a belly-tendon montage. For specific separation electrical potentials were additionally recorded from the abductor pollicis brevis (APB) as well as to the abductor digiti minimi (ADM), the extensor carpi ulnaris (ECU) and the biceps brachii (BB). The EMG signals were amplified and filtered by CED 1902 amplifiers through a CED 1401 power laboratory interface using Spike 2 software (Cambridge Electronic Design, Cambridge, UK).

### Identification of M1 and PMC Stimulation Hotspots

All nTMS examinations were conducted in the hemisphere contralateral to the dominant hand as assessed by the Edinburgh Handedness Inventory. In the first session, nTMS was used to map the primary and pre-motor cortices for a first dorsal interosseus muscle (FDI) stimulation hotspot as previously published (Fleischmann et al., 2013). In brief, M1 was first mapped for an FDI hotspot by definition of the maximal motor evoked potential (MEP) response site with minimal suprathreshold TMS intensity. Subsequently, the PMC was mapped for a hotspot with direct corticospinal output. The hotspot location was validated according to an algorithm that accounts for remote electric field induction in the adjacent M1 when stimulating the PMC. The eXimia system provides the investigator with an estimate of the maximum electric field strength induced under the coil (EF_max_) and at any custom remote site (EF_remote_) (Ruohonen and Ilmoniemi, 1999). It employs a sphere model of the cortical surface to provide dynamic online estimations of the EF_max_ and EF_remote_. This simplified approach has been proven valid in a combined theoretical and stimulation study for targets in cortical stimulation experiments (Thielscher and Kammer, 2002). After PMC hotspot identification, 20 minimal suprathreshold stimuli were applied at an intensity eliciting MEPs with an average amplitude of 200 μV. This confirmed the reliable elicitation of corticospinal volleys. The concomitant EF_remote_ in M1 was then estimated. Subsequently, 20 stimuli were applied with the peak intensity over the M1 hotspot at an intensity sufficient to induce the EF_remote_ previously indirectly induced by PMC stimulation. If no MEPs were elicited by M1 stimulation with that intensity, MEPs elicited by PMC stimulation must have originated in the PMC. Evidence against indirect activation of the corticospinal tract via a PMC-M1 pathway was additionally excluded by the comparison of PMC and M1 MEP onset latencies. MEP onset latencies would be longer than after direct M1 stimulation if indirect activation occurred. Thus, PMC-MEPs of equal or shorter latency provide additional functional evidence for direct activation of corticospinal tracts.

### Motor Task and Virtual Lesion Induction

The 1 mV motor threshold (1 mV-MT) of nTMS over the FDI hotspot was determined for M1 and PMC at the beginning of each experiment. The 1 mV-MT of FDI was defined as the lowest stimulus intensity at which 5 of 10 consecutive stimuli elicited reliable MEP of 1 mV in amplitude in the weakly pre-activated FDI muscle. Pre-activation was induced by the subject holding a pen in the dominant hand as was done later in the motor task.

**Figure 1:**
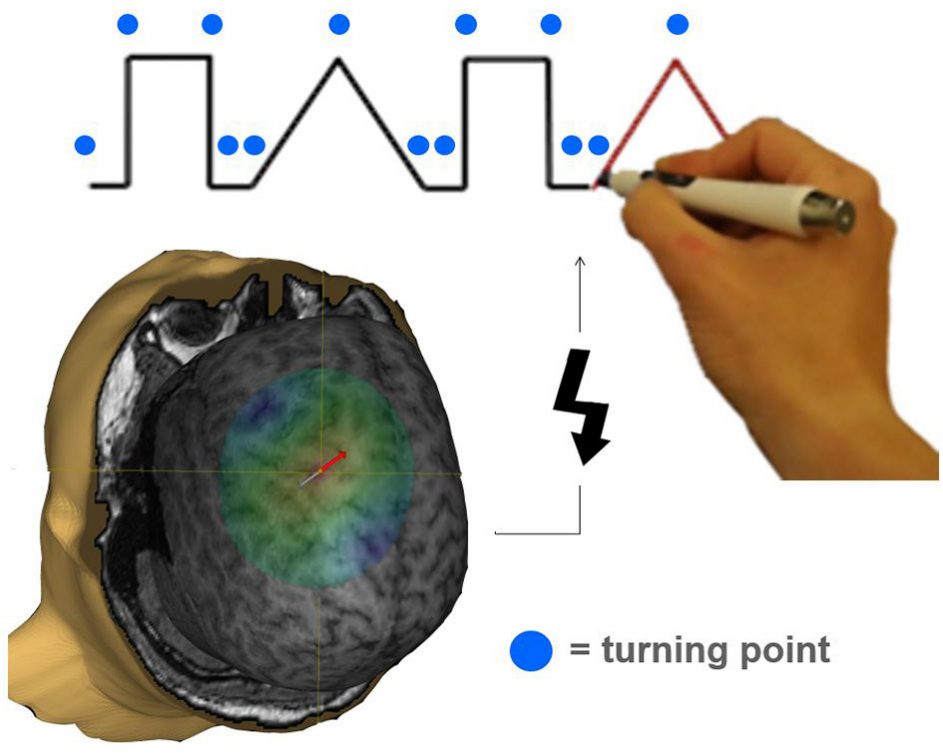
Illustration of a task specifically requiring pre-motor activation during the movement preparation period. The tracking task was adopted from Luria (1966). Participants had to significantly change their movement trajectory at each turning point (indicated by a blue circle). A TMS pulse (indicated by a flash symbol) was applied to M1, PMC, or DLPFC in a randomized order 20, 40, 60, 80, 100, 120, and 140 ms before a turning point occurred interfering with task performance.

During the following experimental task, the EMG electrodes were removed to allow the subject to move the arm unimpeded. The experimental device consisted of a laptop placed directly in front of the subject. The laptop screen depicted a simple combination of two different geometric figures (tri- and rectangles), drawn in one line. Participants were instructed to trace the figures by using a tracking-pen (Figure 1). The pen's coordinates were calculated, sent via infrared and ultrasonic rays to a receiver station (sampling rate 1 kHz) and stored for offline analysis. The tracing pace was predefined by a red line that moved constantly along the track. Guided by this target, it was possible to estimate the position on the track at the time of stimulation. Timing of TMS stimulation was orientated on turning points of the geometric figures. Including two tri- and two rectangles in a randomized sequence, each trial implied 14 changes of direction and therefore a maximum of 14 stimulations per trial. Each run consisted of 20 trials. Per region (M1/PMC/DLPFC), two runs were performed. The six blocks of 20 trials were performed in a randomized sequence. Each trial was started by the subject. After a non-input interval of 30 s the run was terminated automatically. All participants were naïve subjects without any practice.

Single pulses at 1 mV-MT stimulation intensity were delivered randomly at 40, 60, 80, 100, 120, and 140 ms before each turning point. This induced a muscle twitch and a virtual lesion, the sum of which constituted the tracing error. To exclude unspecific effects of frontal lobe TMS, control stimulations of the dorsolateral pre-frontal cortex (DLPFC) ipsilateral to the M1 and PMC under investigation were performed. Stimulation intensity in DLPFC was the same as determined intensity for 1 mV MT in PMC.

### Data Analysis and Statistical Evaluation

Data processing and statistical analysis were carried out using MATLAB (version 2016a, Mathworks, Gatwick, USA).

The application of nTMS at 1 mV-MT intensity inevitably leads to a muscle twitch during execution of the motor task and thus excursion of the pen from the trace to be followed. To investigate whether the muscle twitch is not only equal between M1 and PMC in terms of EMG activity, which was standardized by using 1 mV-MT stimulation intensities, but also with regards to the displacement of the pen, we compared its duration and distance of displacement for all conditions.

Tracking performance was quantified by the error made resulting from the displacement and the return exercise back to the tracking line given as area under the curve (AUC) in mm^2^ starting when the turning point was to be reached and up to 500 ms post turn. Coordinates of the actually drawn line and coordinates of the original tracking line were used to calculate the integral of the difference. In this analysis, a larger AUC indicates that more time was required to correct the interruption, leading to a larger error. Data points were excluded from the evaluation if the subject did not trace the line appropriately (i.e., being 50 ms at given speed ahead or behind the pace) before a turn. To exclude a systemic error due to variable drawing skills between subjects as well as calibration deviation of the tracking-pen, the individual pen's coordinates were offset against the original tracking line.

We used univariate analyses of variance (ANOVA) to test for global effects of main factors, i.e., stimulation site and intervals. If the ANOVA indicated a significant main or interaction effect, *post-hoc* pairwise comparisons were conducted. Alpha error inflation due to multiple comparisons was accounted for by using a Bonferroni correction. Since first-level data on a subject level were not normally distributed (Lilliefors-test), we compared medians within single subjects using the Wilcoxon signed rank test. Median errors among all subjects within the group, however, did show a normal distribution and were therefore compared by Student's paired *t*-Test.

## Results

### Mapping Procedure

There were no adverse effects resulting from the nTMS procedure. Mapping M1 for a stimulation hotspot required on average 133.3 ± 12.6 stimuli. A PMC stimulation hotspot could be identified in all participants after 162.3 ± 20.9 stimuli (mean location 16.3 mm ± 1.65 medial and 18.6 mm ± 1.43 mm anterior to the M1 hotspot). Mean 1mV-MT and thus stimulation intensities for virtual lesion induction were 37 ± 5% maximum stimulator output (MSO) over M1 and 43 ± 8% MSO over PMC and DLPFC, respectively. This difference was not statistically significant (*p* = 0.18).

### No Differential Effect of Suprathreshold nTMS on Pen Displacement During the Task

The duration of pen displacement was not statistically different between M1 (65.71 ms ± 40.9 [range 29–210 ms]) and PMC (64.98 ms ± 48.3 [range 26–280 ms]; *p* = 0.92). Furthermore, the extent of displacement was also comparable between M1 (14.72 mm ± 22.78 [range 1–116.2 mm]) and PMC (13.19 mm ± 24.09 [range 1–193.17]; *p* = 0.69). No visible muscle twitch was induced by DLPFC stimulation.

### Virtual Lesion Effect of nTMS on Task Performance

**Figure 2:**
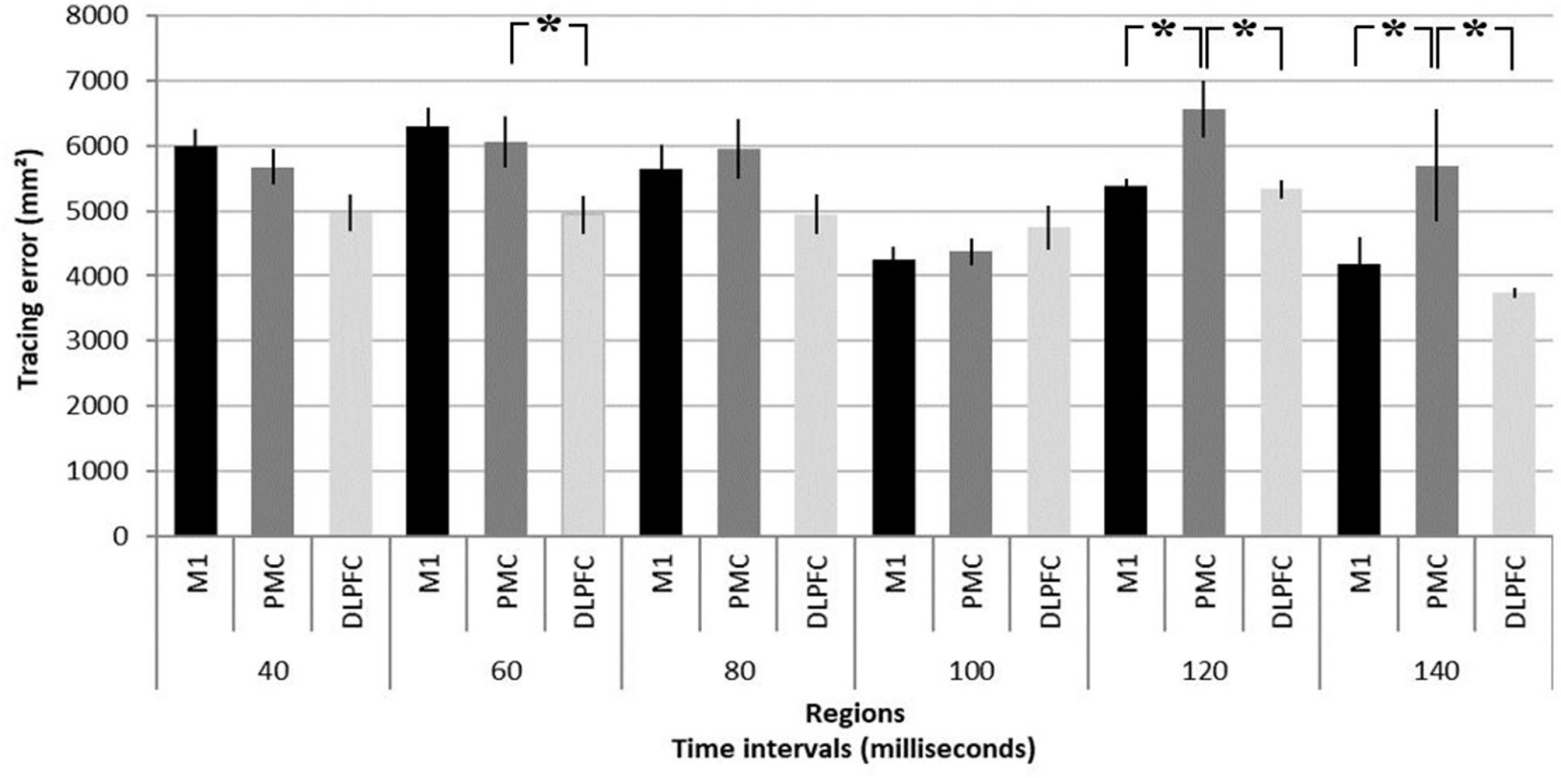
Summary of virtual lesion effects on task performance. Tracing error as response to virtual lesions induced in the primary motor area (M1), dorsal pre-motor area (PMC), and dorsolateral pre-frontal cortex (DLPFC) at time intervals of 40, 60, 80, 100, 120, and 140 ms before participants had to significantly change their arm movement trajectory in a motor task (see Figure 1 for task description). It should be noted that the tracing error resulting from a pre-motor virtual lesion at 120 and 140 ms is significantly larger than in M1 and DLPFC. *p < 0.05. Error bars denote standard deviations.

**Figure 3:**
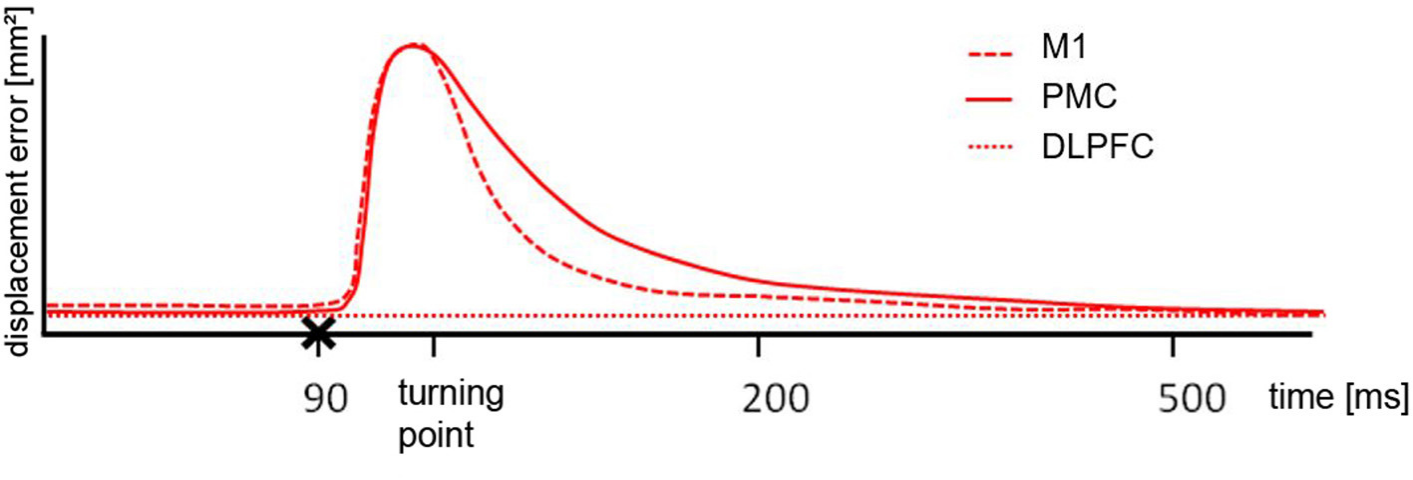
Schematic illustration of the results. After cortical motor area stimulation (x) from 70 ms pre-turn until the turning point the error size was different to the DLPFC but the comparable between M1 and PMC, indicating a similar deflection. From turning point until 200 ms post turn the difference for M1 and PMC stimulation was significantly different. For the period of 200 ms until 500 ms post turn there was no difference between any of the stimulated areas.

NTMS over different regions differently affected performance [*F*_(2, 20)_ = 7.79, *p* = 0.004]. Results are summarized in Figure 2 and illustrated in Figure 3. NTMS-induced virtual lesions of the PMC influenced performance (mean 5722.26 mm^2^ ± 209.17) more significantly than DLPFC TMS (4778.23 mm^2^ ± 120.76; *p* = 0.000) and tended to be more than M1 (5287.07 mm^2^ ± 160.72, *p* = 0.068).

Stimulation intervals significantly influence task performance [*F*_(5, 40)_ = 9.12, *p* = 0.000] and importantly show an interaction effect with regions [*F*_(10, 80)_ = 3.25, *p* = 0.001]. Virtual lesions of PMC induce significantly larger errors compared to M1 and DLPFC stimulation at 120 ms (6563.14 mm^2^ ± 427.30 vs. M1: 5376.84 mm^2^ ± 124.13, *p* = 0.021; DLPFC 5331.46 mm^2^ ± 139.47, *p* = 0.016) and 140 ms (5698.03 mm^2^ ± 857.35 vs. M1: 4178.08 mm^2^ ± 411.50, *p* = 0.003; and DLPFC 3742.18 mm^2^ ± 75.12, *p* = 0.000). Finally, PMC stimulation led to larger errors at 60 ms as compared to stimulation of the DLPFC (6076.75 mm^2^ ± 364.35 vs. 4958.08 mm^2^ ± 312.50, *p* = 0.013).

Neither stimulation intensity (*β* = −0.0074, *p* = 1) nor stimulation order (i.e., if PMC was first or not) did influence results at 120 ms [*F*_(2, 8)_ = 0.11, *p* = 0.9] or 140 ms [*F*_(2, 8)_ = 0.28, *p* = 0.76].

## Discussion

We were able to replicate previous findings that pre-motor corticospinal projections are directly connected to hand muscles and can thus be reliably identified in humans through nTMS. We extend on previous findings by elucidating their role for human motor behavior. Virtual lesions of pre-motor corticospinal projections not only resulted in performance errors comparable to virtual lesions of more dense M1 corticospinal projections, but induced tracking errors were even higher and can be related to errors in spatio-temporal integration. This provides novel evidence that pre-motor activation is crucial for task performance early in the stage of motor preparation in humans, suggesting a significant role shaping voluntary movement. Results are consistent with a hierarchical model of motor control and their temporal pattern is similar to that observed in primates.

### Validity of the Design for Virtual Lesions of Investigated Regions

The present study shows that when applied over the PMC contralateral to the dominant hand, online single pulse TMS can affect the correction time needed to perform a motor preparation exercise. Compared to the control site (DLPFC) the observed error after PMC and M1 stimulation was significantly larger. Since M1 is a major source of descending projections to motor neurons (Dum and Strick, 1991) and responsible for motor execution, the measured error is supposed to occur mainly due to muscle activation induced by suprathreshold stimulation. Evaluations of twitch-induced displacements of the pen, however, clearly show that these are not different M1 and PMC. The difference between M1 and PMC stimulation thus indicates an additional disruption of motor planning processes in PMC by inducing a virtual lesion. Some unspecific error was seen when the stimulus was applied to the DLPFC with the same intensity as used over PMC. Chosen as control site because not supposed to be involved in motor control in this task, the error measured during stimulation over DLPFC could display a basic error level.

Previous studies that proved the significance of the PMC for motor planning chose experimental designs based on reaching and grasp movement (Davare et al., 2008; Baumer et al., 2009; Busan et al., 2009). Precision grasping task and the present experimental designs are comparable insofar as both comprise a visually guided motor planning task which has to be performed with upper limbs. Since the present results are consistent with these previous findings, they confirm the used algorithm for PMC hotspot examination. Yet, none of the studies conducted targeted investigations of corticospinal projections. They furthermore did not interfere with an ongoing task and our study is the first investigation to include error correction capabilities during motor planning. Regarding error correction, it must be duly noted that the pace of the tracking task was intentionally high to inevitably cause tracing errors that need to be corrected for, even in the control condition. Hence, investigation of error correction capabilities were inherent to the study design and not only induced by displacement of the pen through muscle twitches, since larger error were also seen following control stimulations not eliciting muscle twitched (i.e., DLPFC stimulation).

### Comparison of Pre-motor Disruptive Virtual Lesion Latencies to Previous Studies

Only after application of virtual lesions 120–140 ms before the trajectory of movement had to be adjusted, an effect on task performance was evident. To our knowledge, there is no other study that investigated pre-motor corticospinal projections through a virtual lesion during an ongoing motor task. Previous studies used reaction time tasks, which investigate more global functions of the pre-motor area. Importantly, the role of the PMC in correcting errors between actual and desired movements has not been investigated before (Macintyre et al., 2018).

Previous studies were able to delay responses during reaction-time tasks by stimulating the M1 and the PMC after cue presentation (Schluter et al., 1998). Thereby the performance was influenced at short cue-stimulus intervals of 100–140 ms when the stimulus was applied over PMC and at longer cue-stimulus intervals of 300–340 ms when applied over M1. Schluter et al. measured these time intervals from the onset of a cue which appeared on a screen. In the current study, there was no analog onset of the performance due to the experimental design what makes it difficult to compare the results of the two studies. Because all four turning points were visible from the beginning of one trial, subjects had plenty of time to prepare for the task. The results indicate that 120–140 ms pre-turn the PMC seems to be in a processing phase to perform the task. This period of motor planning could be comparable with the interval of 100 ms post cue presentation, confirming the observations of Schluter et al.

Given that PMC stimulation was applied with supra-threshold intensity, the elicitation of a motor evoked potential (MEP) is possible via both corticocortical and corticospinal pathways. While Cattaneo et al. found evidence for a cortico-cortical mechanism that mediates object-driven grasp (Cattaneo et al., 2005), Teitti et al. and our group could elicit MEPs in the hand muscles directly from NPMAs (Teitti et al., 2008; Fleischmann et al., 2013). As mentioned above, according to Schluter et al. the executive processing by M1 takes place several milliseconds later than planning processes in PMC. This suggests an information flow from anterior (premotor) to posterior (primary motor) cortical regions during the processing period. Considering that in the present study PMC was interrupted by stimulating 120–140 ms pre-turn, there should be an effect on error size when M1 is stimulated several milliseconds later. However, task performance was not impaired after M1 stimulation compared to PMC stimulation at shorter stimulus intervals. This indicates that effects occuring after PMC stimulation are not due to cortical connections via M1, but rather to subcortical or direct corticospinal projections. The fact that during PMC mapping the focus was directed to corticospinal subpopulations supports this finding. Further experiments with shorter as well as extended stimulus intervals would be necessary to confirm this idea and determine time intervals of M1 and PMC processing in this specific experimental design.

As previously mentioned, also Busan et al. interfered with an experimental performance by applying TMS during a motor planning task. They found that the reaction time during reaching task performance was not prolonged but shortened after single pulse TMS over PMC. This contrary effect was observed when the stimulus was applied after the onset but before starting the task at 75% of mean reaction time (approximately at 150 ms). By using a lower stimulation intensity of 110% and applying the stimulus much earlier, these data might differ from present findings due to a pre-activation of the area.

The relation of effect and time of stimulation was shown in previous studies which found facilitatory effects when single-pulse was applied shortly before (Topper et al., 1998; Grosbras and Paus, 2003) and disruptive effects when applied during a cognitive process (Walsh and Rushworth, 1999). These observations confirm the assumption that results of the present study are due to an interfering effect by inducing a virtual lesion during the performance processing in PMC, whereas Busan et al. obtained an opposite and rather beneficial effect by an earlier priming stimulation.

### Possibility of Remote Effects

Stimulation intensities were not significantly different between stimulation sites and subjects, which renders a systemic error through different stimulation intensities unlikely. However, it is possible that the current spreads from the PMC corticospinal stimulation hotspot to surrounding pre-motor areas, and thus the effect is unspecific for PMC projections under investigation (Schmidt et al., 2015). Although this possibility can indeed not be excluded, we believe that the experimental design should decrease the probability of this error to some extent. Importantly, all subjects received virtual lesions rostral and caudal to the PMC stimulation site and thus unsystematic remote stimulations of pre-motor areas could have equally likely occurred from stimulation of the DLPFC and M1 (Fleischmann et al., 2020). Furthermore, the use of nTMS is shown to provide a resolution of <2 mm, which should not have caused systematic spread of electric fields to adjacent pre-motor areas. Finally, the use of a monophasic figure-of-eight coils allows for superior directionality of the stimulation compared to biphasic coils (Ilmoniemi et al., 1999).

### Limitations

As proven by several studies, repetitive TMS can change the excitability of stimulated neurons as well as remote areas (Rizzo et al., 2004). During 90 min of experimental performance, as carried out in the present study, the stimulation might have had an rTMS character and therefore influenced the excitability of cortical areas. However, by keeping short breaks of several minutes in between the trials and a relative long interstimulus interval of some 100 ms, the stimulation was no repetitive form. In addition Civardi et al. showed that even single pulse TMS at low-intensity applied over pre-motor areas can engage corticocortical connections to M1 (Civardi et al., 2001). However, the conditioning stimuli had no effect on responses evoked in the active, but only in the relaxed FDI muscle. Therefore, potential excitability changes due to single pulse TMS should not have any influences on the current results.

Another disadvantage of the chosen experimental design is the inexplicable high error level after stimulation of DLPFC. As mentioned above, this area displays the baseline error because it is assumed not to be involved in motor performance. Therefore, the measured error might be systematic. The pace of the tracking task was indeed set high to force the system to work at its limits and yield tracing errors that need to be corrected for. Thus, yielding high errors in the control condition was desired and should not be regarded as virtual lesion effect of DLPFC stimulation. Nonetheless, previous studies showed that intracortical interactions between the DLPFC and primary and secondary motor areas can be expected (Hasan et al., 2013). Since errors following DLPFC stimulation were highest in the very early stimulation intervals (i.e., 30 ms), we think that long-range intracortical mechanisms should be negligible in this context.

It is well-established that pre-activation of target muscles causes changes in the intracortical micro-circuitry, including surround inhibition of neighboring muscles, which is affected by physical parameters or constraints such as joint angles at the time of stimulation. This is ideally controlled for in an experimental setup. We were, unfortunately, unable to control for these covariates since this would have required high-frequency three-dimensional tracking of joints involved in the task. We rather argue that due to the vast number of trials, randomized order between trials and equal conditions over all regions studied this error should be systematic and not favor any of the regions.

## Conclusion

Pre-motor corticospinal projections can be reliably identified and their role for human motor behavior extends on primary motor projections by early involvement in motor preparation and error correction of an ongoing movement. This interpretation is supported by the observation of complex movements induced by direct cortical stimulation in the pre-motor area and higher-order motor deficits after pre-motor lesions. These results should be considered in rehabilitation of lesions that include the pre-motor area and when dexterity remains impaired despite apparent recovery of corticospinal function. On the other hand, non-invasive brain stimulation studies should investigate pre-motor corticospinal projections as target to enhance recovery beyond simple corticospinal integrity.

## Data Availability Statement

The raw data supporting the conclusions of this article will be made available by the authors, without undue reservation.

## Ethics Statement

The studies involving human participants were reviewed and approved by Ethikkommission der Charité - Universitätsmedizin Berlin, Campus Charité Mitte, Charitéplatz 1, 10117 Berlin. The patients/participants provided their written informed consent to participate in this study.

## Author Contributions

RF, PT, and SHS conceived and planned the experiments. RF and SHS carried out the experiments and took the lead in writing the manuscript. All authors contributed to the interpretation of the results, provided critical feedback and helped shape the research, analysis, and manuscript.

## Conflict of Interest

The authors declare that the research was conducted in the absence of any commercial or financial relationships that could be construed as a potential conflict of interest.
